# USP8 positively regulates hepatocellular carcinoma tumorigenesis and confers ferroptosis resistance through β-catenin stabilization

**DOI:** 10.1038/s41419-023-05747-7

**Published:** 2023-06-13

**Authors:** Jianing Tang, Guo Long, Liang Xiao, Ledu Zhou

**Affiliations:** 1 Department of Liver Surgery, Xiangya Hospital, Central South University, Changsha, 410008 Hunan China; 2 National Clinical Research Center for Geriatric Disorders, Xiangya Hospital, Central South University, Changsha, Hunan 410008 China

**Keywords:** Cancer, Cell death

## Abstract

Hepatocellular carcinoma (HCC) is the most common type of primary hepatic carcinoma, which is a growing public health problem worldwide. One of the main genetic alterations in HCC is the deregulated Wnt/β-catenin signaling, activation of β-catenin is associated with the progression of HCC. In the present study, we aimed to identify novel modulators in controlling β-catenin ubiquitination and stability. USP8 was overexpressed in HCC tissues and correlated with β-catenin protein level. High expression of USP8 indicated poor prognosis of HCC patients. USP8 depletion significantly decreased β-catenin protein level, β-catenin target genes expression and TOP-luciferase activity in HCC cells. Further mechanistic study revealed that the USP domain of USP8 interacted with the ARM domain of β-catenin. USP8 stabilized β-catenin protein via inhibiting K48-specific poly-ubiquitination process on β-catenin protein. In addition, USP8 depletion inhibited the proliferation, invasion and stemness of HCC cells and conferred ferroptosis resistance, which effects could be further rescued by β-catenin overexpression. In addition, the USP8 inhibitor DUB-IN-3 inhibited the aggressive phenotype and promoted ferroptosis of HCC cells through degradation of β-catenin. Thus, our study demonstrated that USP8 activated the Wnt/beta-catenin signaling through a post-translational mechanism of β-catenin. High expression of USP8 promoted the progression and inhibited ferroptosis of HCC. Targeting the USP8 may serve as a promising strategy for patients with HCC.

## Introduction

Liver cancer is one of the most urgent public health problems worldwide, which is the sixth most common neoplasm and is the fourth leading cause of cancer-related deaths in all tumors worldwide [1]. Hepatocellular carcinoma (HCC) originates predominantly from hepatocytes, it is the most common histological type of primary hepatic carcinoma, accounting for approximately 90% of primary liver cancers. HCC is known as its high malignance, rapid progression, high metastatic potency, easy recurrence, poor clinical outcomes and is usually indetectable [2]. Despite the developments in major clinical interventions, including surgery, transplantation, chemotherapy, radiotherapy, drug-targeted therapy, and immunotherapy. The advanced stage of HCC is still a therapeutic challenge, and the overall 5-year survival rate of patients with HCC is less than 20% [3–5]. Therefore, it is an urgent need to identify the novel molecular markers and develop effective approaches to treat patients with HCC.

Wnt/β-catenin pathway is a highly conserved pathway that mainly controls cell proliferation. The activation of Wnt/β-catenin pathway is associated with haematopoietic system development, hair follicle renewal, liver metabolism and regeneration, lung tissue repair and metabolism, and the maturation of osteoblast [6–9]. This pathway comprises the following segments: the extracellular signal; trans-membraned segment; cytoplasmic compounds including glycogen synthase kinase 3 β (Gsk3β), adenomatous polyposis coli (Apc), Axin/conductin, casein kinase1α (CK1α), and disheveled (Dvl in mammals and Dsh in drosophila); the nuclear segment mainly contains β-catenin and TCF/LEF family members [10]. The activation of Wnt/β-catenin pathway is mediated by the binding of extracellular Wnt ligands to membrane receptors through autocrine/paracrine methods. β-catenin functions as the ultimate effector of Wnt/β-catenin pathway [11, 12]. After receiving the upstream activation signals, intracellular β-catenin is rapidly accumulated. Then β-catenin is trans-localized into the nucleus and binds to TCF/LEF family members to drive transcription of Wnt/β-catenin target genes involved in cell survival, differentiation, proliferation, and migration [13].

At the off state of Wnt/β-catenin signalling pathway, β-catenin binds to the cytoplasmic sides of cadherin and a degradation complex (DC) comprising APC, AXIN, CK1 and GSK3 protein is formed. β-catenin is captured and phosphorylated by DC, thus activating the ubiquitin proteasome-mediated degradation of β-catenin and keep the low level of free β-catenin in the cytoplasm [14]. Abnormal activation of the Wnt/β-catenin pathway is closely related to various cancers. Almost all colon cancers are associated with mutations in APC, AXIN2, or β-catenin [15]. The mutations of β-catenin are also frequently observed in HCC, which are missense and enhance the stabilization and unrestrained transcriptional activity of β-catenin protein by inhibiting its phosphorylation and degradation [16]. In HCV-related HCCs, growing evidence suggests that mutations and overexpression of β-catenin occur more frequently. Activation of the Wnt/β-catenin cascade is associated with the stemness, drug resistance, progression, and metastasis of HCC [17–21]. Indeed, Wnt/β-catenin pathway promotes self-renewal and in vivo tumorigenicity of HCC cancer stem cells (CSCs) [22, 23]. Furthermore, the activation of Wnt/β-catenin is closed related to the resistance to regorafenib and sorafenib in HCC patients [24]. The protein stability of β-catenin is mainly regulated by phosphorylation and ubiquitination. While the deubiquitination and stabilization function of deubiquitinating enzymes (DUB) responsible for β-catenin remains largely unknown. Therefore, it is important to identify the potential DUBs in controlling β-catenin stabilization which can be exploited for therapeutic interventions.

In the present study, we report that a deubiquitinating enzyme, USP8, regulates HCC cells proliferation, invasion, stemness and ferroptosis resistance via the Wnt/β-catenin pathway. Mechanistically, USP8 deubiquitinates and stabilizes β-catenin in a catalytic activity-dependent manner. Furthermore, USP8 is overexpressed HCC samples, suggesting that USP8 may play a role in the pathogenesis of HCC.

## Results

### USP8 depletion inhibits Wnt/β-catenin signaling pathway activity

**Fig. 1:**
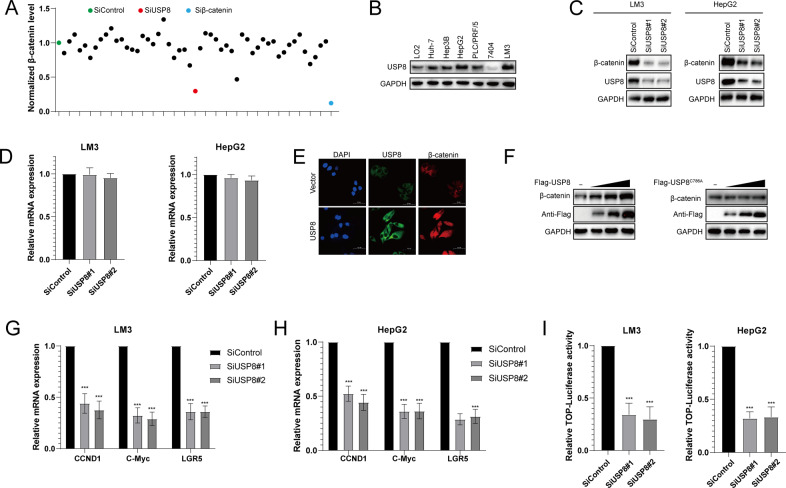
USP8 depletion decreases Wnt/β-catenin signaling activity in HCC cells. **A** The siRNAs specific to each deubiquitinating enzyme were transfected into LM3 cells. After 48 h, cells were lysed and the β-catenin protein level was analyzed by Western blot. **B** Western blot analysis of USP8 protein abundance in HCC cell lines and normal liver cells. **C**, **D** USP8 depletion decreased β-catenin protein level without affecting mRNA expression of β-catenin. **E** Immunofluorescence assay of USP8 and β-catenin. **F** Increasing amounts of USP8 WT or C786A were transfected into LM3 cells and β-catenin expression was detected. **G**, **H** USP8 depletion decreased β-catenin target genes using two different siRNA oligos. **I** USP8 depletion decreased TOP-luciferase activity. LM3 and HepG2 cells were transfected with SiUSP8 or SiControl together with TOP-luciferase reporter plasmid. Luciferase activity was measured 48 h after transfection. The results shown are representative of 3 independent experiments. Data are represented as mean ± SD of biological triplicates *P-value < *0.05*; **P -value < *0.01*; ***P-value < *0.001* by unpaired, 2-tailed Student’s t-tests.

**Fig. 2:**
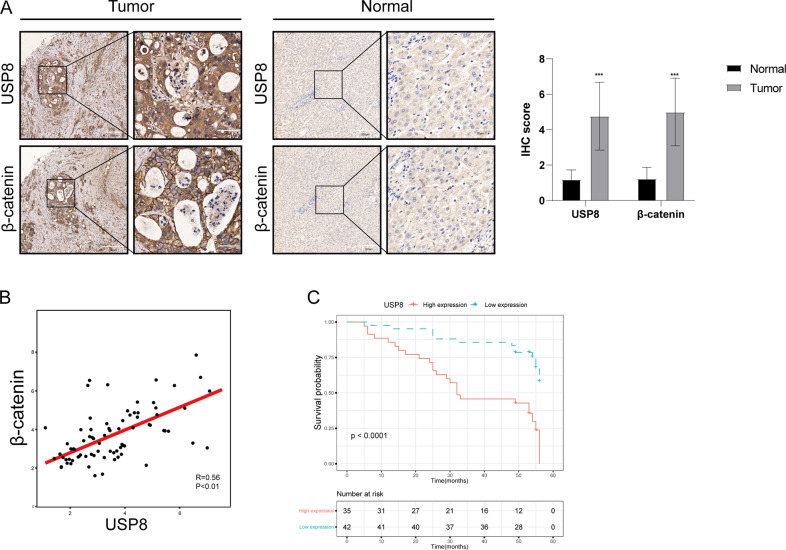
USP8 correlates with β-catenin protein levels in human HCCsamples. **A** USP8 and β-catenin was upregulated in HCC. 97 samples were used for IHC analysis, including normal liver tissue (*n* = 20) and HCC tissues (*n* = 77). Specific primary antibodies against β-catenin (Proteintech, 17565-1-AP), USP8 (Proteintech, 67321-1-Ig) were used for IHC. **B** USP8 correlated β-catenin in human HCC samples. **C** High expression of USP8 was associated with poor prognosis.

To find potential deubiquitinating enzymes that could regulate Wnt/β-catenin signaling pathway in HCC, we screened a DUB siRNA library (The Human ON-TARGETplus Deubiquitinating Enzyme siRNA Library, Dharmacon, Gu-104705). Briefly, we transfected four nonoverlapping siRNA mixtures specific for each of the DUBs into LM3 cells and found that silencing USP8 significantly decreased β-catenin protein level in LM3 cells (Fig. 1A). We tested the expression of USP8 in HCC cell lines (Huh-7, Hep3B, HepG2, PLC/PRF/5, 7404, LM3) and normal liver cell line (LO2), we observed that USP8 was highly expressed in HepG2 and LM3 cells (Fig. 1B). To further investigate the function of USP8 in regulating β-catenin protein level, two non-overlapping siRNAs targeting USP8 were transfected separately in LM3 and HepG2 cells (Fig. 1C). RT-PCR analysis indicated that USP8 did not change the mRNA abundance of β-catenin (Fig. 1D). Immunostaining results indicated that β-catenin was upregulated and translocated from cytosol to the nuclei after ectopic expression of USP8 (Fig. 1E). Consistently, overexpression of the wild-type USP8 significantly increased the expression of β-catenin in a dose-dependent manner. While the catalytically inactive mutant C786A (USP8^C786A^) could not increase β-catenin protein levels, indicating that USP8 increased β-catenin protein level depending on its DUB activity (Fig. 1F). We then evaluated the expression of β-catenin target genes (CCND1, c-MYC and LGR5) and observed that knockdown of USP8 inhibited the transcription of CCND1, c-MYC and LGR5 (Fig. 1G, H). To further determine whether USP8 influenced the transcriptional activity of β-catenin, we examined TOP-luciferase reporter gene activity by USP8 depletion. Our results indicated that USP8 depletion decreased TOP-luciferase reporter gene activity in LM3 and HepG2 cells (Fig. 1I). IHC analysis indicated that USP8 and β-catenin were both upregulated in HCC samples (Fig. 2A). Besides, we observed a positive correlation between USP8 and β-catenin protein levels in human HCC samples (Fig. 2B). Survival analysis revealed that high expression of USP8 was related to poor prognosis (Fig. 2C, Table 1). Taken together, these results indicated that USP8 was a regulator of Wnt/β-catenin signaling pathway and was a prognostic marker.

**Table 1:** Clinicopathologic characteristics of HCC patients according to the expression of USP8.

Variables	USP8		*P*-value
	High Expression	Low Expression	
Gender			0.244
Female	5	4	
Male	30	38	
Age at diagnosis (years)			0.488
≤ 50	13	20	
> 50	22	22	
T stage			0.132
T1	22	32	
T2	11	10	
T3	2	0	
N stage			0.873
N0	32	38	
N1	3	4	
Grade			0.727
I	2	2	
II	25	27	
III	8	13	
Number of tumors			0.534
1	32	37	
2	3	4	
3	0	1	
Number of cirrhotic nodules			0.362
0	0	1	
1	1	5	
2	21	21	
3	13	15	
Stage			0.247
I	22	32	
II	12	10	
II	1	0	
Vital status			< 0.001
Alive	9	30	
Dead	26	12	

### USP8 stabilizes β-catenin through the Ub-Proteasome pathway

**Fig. 3:**
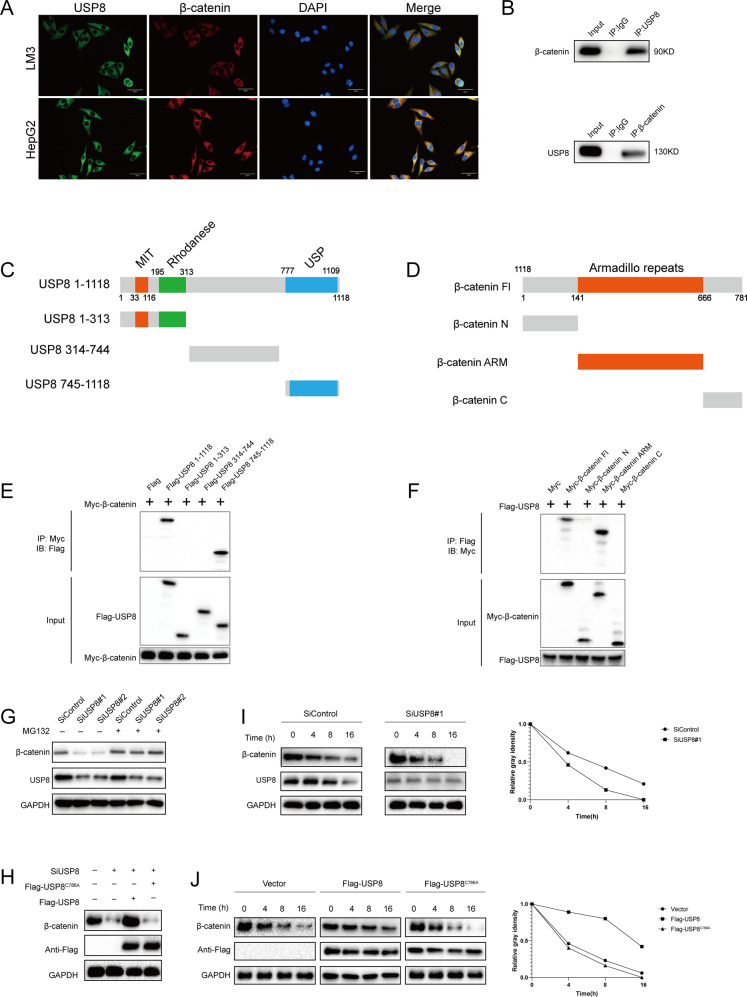
USP8 associates with β-catenin and increases its stability. **A** An immunofluorescence assay demonstrated that USP8 and β-catenin at least partially colocalized in LM3 and HepG2 cells. **B** Co-IP assay reveals association between endogenous USP8 and β-catenin in LM3 cells. LM3 cells were harvested with RIPA lysis buffer. Co-IP was performed using antibody as indicated. **C**, **D** USP8 and β-catenin domain structure and deletion mutants used in the study. **E** The USP domain of USP8 interacted with β-catenin HEK293 cells were transfected with 2 µg Myc-β-catenin together with Flag-USP8 full-length or mutants. After 24 h, cells were harvested with NP-40 lysis buffer. Co-IP was performed using Myc antibody. The possible interacted USP8 domains were detected by Flag antibody. **F** The ARM domain of β-catenin interacted with USP8. HEK293 cells were transfected with 2 µg Flag-USP8 together with Myc-β-catenin full length or mutants. After 24 h, cells were harvested with NP-40 lysis buffer. Co-IP was performed using Flag antibody. The possible interacted β-catenin domains were detected by Myc antibody. **G** LM3 cells transfected with the indicated siRNA were treated with or without the proteasome inhibitor MG132 (10 µM, 6 h), and then proteins were analyzed. **H** USP8 WT or C786A was introduced into LM3 cells together with USP8 siRNA. β-catenin levels were measured. **I** LM3 cells transfected with USP8 siRNA were treated with cycloheximide (10 µg ml^−1^), and collected at the indicated times for western blot. Quantification of β-catenin levels relative to GAPDH is shown. **J** Half-life analysis of β-catenin in LM3 transfected with the indicated plasmids. Results shown are representative of 3 independent experiments. Data are represented as mean ± SD of biological triplicates *P-value < *0.05*; **P-value < 0.01, ***P-value < *0.001* by unpaired, 2-tailed Student’s t-tests.

We then performed immunofluorescence assay to assess cellular localization of USP8 and β-catenin. Immunostaining results indicated that β-catenin and USP8 colocalized both in the cytosol of HCC cells (Fig. 3A). Co-immunoprecipitation assay indicated the direct association between USP8 and β-catenin under physiological conditions. Additionally, deletion analysis revealed that the USP domain of USP8 physically interacted with the ARM domain of β-catenin (Fig. 3C–F). These findings indicated that USP8 and β-catenin formed an intact complex. Since USP8 interacted with β-catenin and USP8 depletion decreased β-catenin levels, we hypothesized that USP8 may regulate the turnover of β-catenin through the Ub-proteasome pathway. Depletion of USP8 by two non-overlapping siRNAs separately markedly decreased β-catenin levels. While in the presence of MG132, β-catenin protein levels were not affected by USP8, indicating that USP8 regulated β-catenin degradation through ubiquitin-proteasome pathway. Consistently, the decrease of β-catenin induced by USP8 depletion could be reversed by overexpression wild-type (WT) USP8, but not its catalytically inactive mutant (USP8-C786A) (Fig. 3G, H). To prove that USP8 affects β-catenin stability, cycloheximide (CHX) were used to treat LM3 cells for 0, 4, 8, and 16 h. The half-life of β-catenin was shortened in cells depleted of USP8. And β-catenin stability was increased upon overexpression USP8-WT but not USP8-C786A (Fig. 3I, J). These results indicated that USP8 stabilizes β-catenin in cells through the Ub-proteasome pathway.

### USP8 deubiquitylates β-catenin

**Fig. 4:**
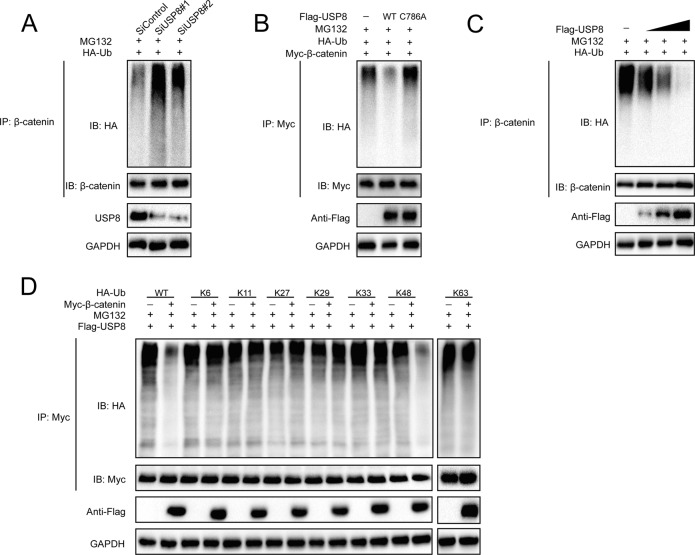
USP8 de-polyubiquitylates β-catenin. **A** LM3 cells transfected with the indicated siRNA were treated with MG132 for 6 h before collection. β-catenin was immunoprecipitated with anti-β-catenin and immunoblotted with anti-HA. **B** Immunoblotting was used to detect the ubiquitination of β-catenin in 293 T cells co-transfected with Myc-β-catenin, HA-Ubiquitin, and Flag-USP8 (wild type or C786A). **C** USP8 removed the ubiquitin chain of β-catenin in a dose-dependent manner. **D** HA-WT, K6, K11, K27, K29, K33, K48 or K63 Ub were co-transfected with Myc-β-catenin and Flag-USP8 into HEK293T cells. After treatment with 10 μM MG132 for 6 h, cell lysates were subjected to ubiquitination assay and the ubiquitination level of β-catenin detected by HA antibody.

Since USP8 is a deubiquitylase, we went on to examine whether β-catenin is a substrate of USP8. As illustrated in Fig. 4, the knockdown of USP8 profoundly increased the ubiquitin level of β-catenin. Conversely, ectopic expression of USP8-WT, but not USP8-C786A, significantly inhibited the ubiquitylation of β-catenin in cells (Fig. 4A, B). In vivo ubiquitylation assays showed that USP8 reduced the ubiquitin level of β-catenin in a dose-dependent manner (Fig. 4C). To further investigated which type of ubiquitin chain of β-catenin was removed by USP8, we further performed ubiquitination assay with different kinds of mutant ubiquitin (K6, K11, K27, K29, K33, K48, and K63). The result indicated that USP8 could efficiently remove the K48-linked ubiquitin chain from β-catenin protein (Fig. 4D). These results suggested that USP8 may act as a β-catenin-directed DUB which deubiquitylated and stabilized β-catenin.

### USP8 regulates cell proliferation, invasion, stem-like properties, and ferroptosis through β-catenin

**Fig. 5:**
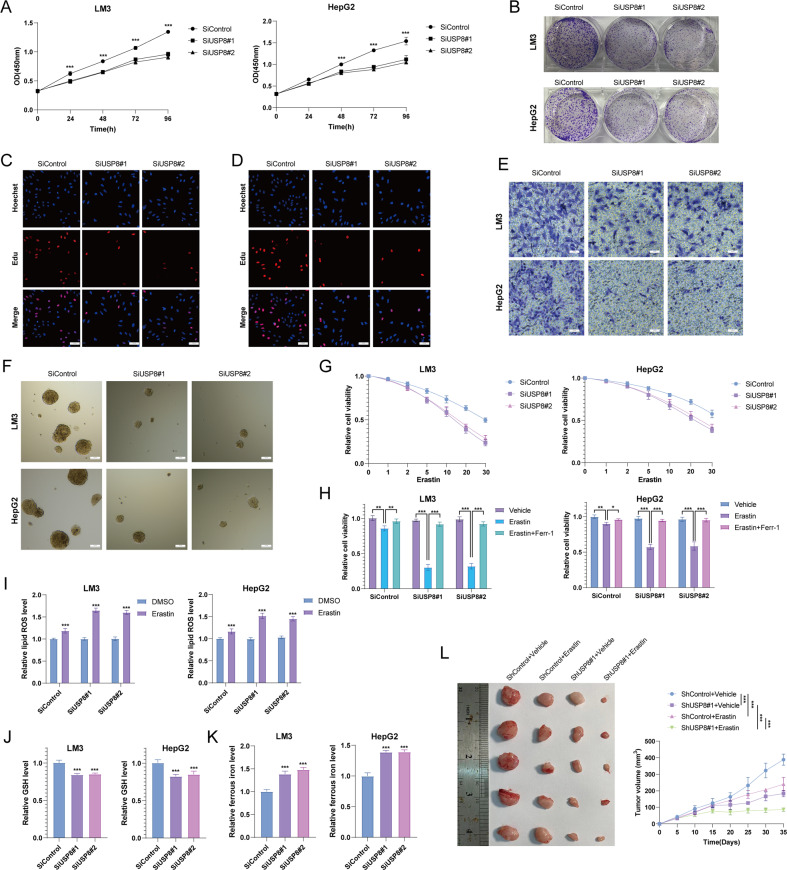
USP8 depletion inhibits HCC cell proliferation, invasion stem-like properties and promotes ferroptosis. **A** USP8 depletion inhibited HCC proliferation. **B** USP8 depletion decreased clone formation capability of HCC cells. **C**, **D** Representative images of EdU assay of HCC cells. **E** Tranwell invasion assay of HCC cells. **F** USP8 depletion decreased sphere formation of HCC cells. **G** CCK8 assay was used to analyze the responses of HCC cells to erastin. **H** CCK8 assay showing the response of LM3 and HepG2 cell lines to erastin (20 μM)/± ferrostatin-1 (1 μM). **I** Lipid ROS, (**J**). ferrous iron levels, (**K**) and GSH levels were measured in LM3 cells and HepG2 cells. **L** USP8 depletion inhibits the tumor growth in vivo. LM3 cells were stably transfected with lentivirus carrying a scrambled shRNA or USP8 shRNA. 1 × 10^6^ LM3 cells were injected to the right dorsal flank of each mouse. Tumor sizes were measured every 3 days until the end of the experiment. The results shown are representative of 3 independent experiments. Data are represented as mean ± SD of biological triplicates *P-value < *0.05*; **P-value < *0.01*; ***P-value < *0.001* by unpaired, 2-tailed Student’s t-tests.

**Fig. 6:**
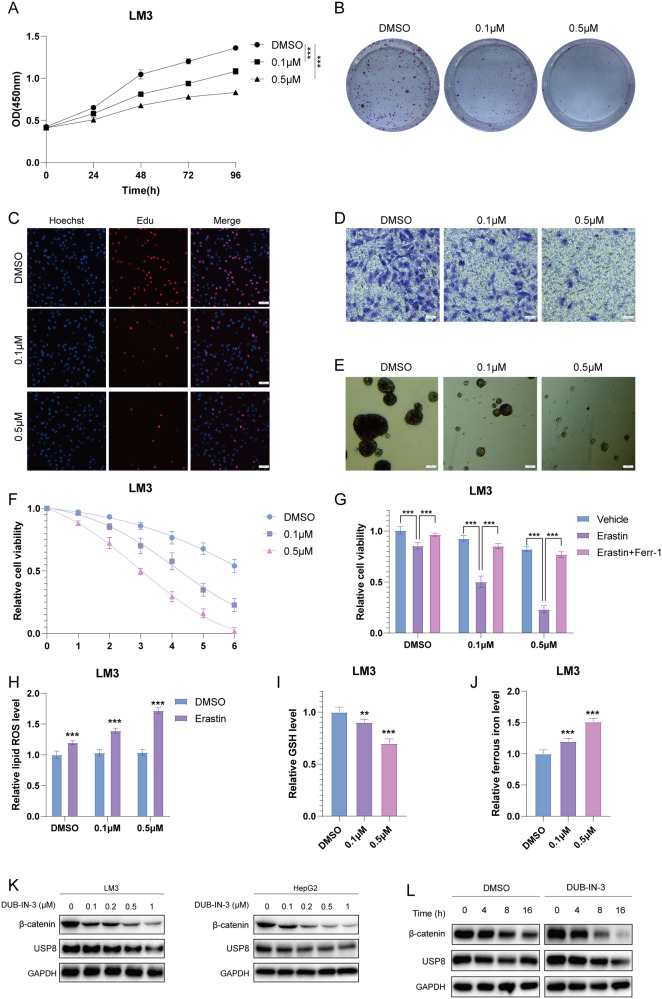
DUB-IN-3 inhibits HCC cell proliferation, invasion, stem-like properties and promotes ferroptosis. **A** DUB-IN-3 inhibited cell proliferation in LM3 cells. **B** DUB-IN-3 decreased clone formation capability of LM3 cells. **C** Representative images of EdU assay of LM3 cells. **D** Transwell migration assay of LM3 cells. **E** Sphere formation assay of LM3 cells. **F** CCK8 assay was used to analyze the responses of LM3 cells to erastin. **G** CCK8 assay showing the response of LM3 cells to erastin (20 μM)/± ferrostatin-1 (1 μM). **H** Lipid ROS, (**I**). ferrous iron levels, (**J**). and GSH levels were measured in LM3 cells and HepG2 cells. **K** DUB-IN-3 reduced β-catenin protein level. **L** DUB-IN-3 treatment decreased the half-life of β-catenin. Results shown are representative of 3 independent experiments. Data are represented as mean ± SD of biological triplicates *P-value < *0.05*; **P-value < *0.01*; ***P-value < *0.001* by unpaired, 2-tailed Student’s t-tests.

**Fig. 7:**
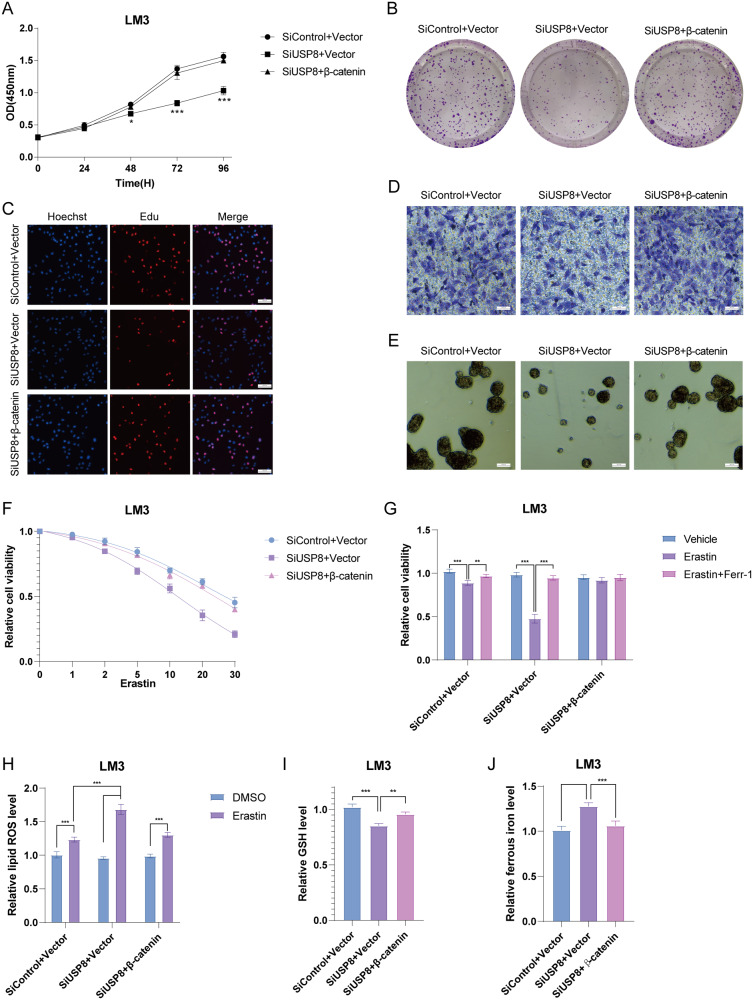
Increased β-catenin expression reverses the effect induced by USP8 depletion. **A** Cell proliferation assay of LM3. **B** Clone formation assay of LM3. **C** Representative images of EdU assay of LM3. **D** Transwell invasion assay of LM3. **E** Sphere formation assay of LM3. **F** CCK8 assay was used to analyze the responses of LM3 cells to erastin. **G** CCK8 assay showing the response of LM3 cells to erastin (20 μM)/± ferrostatin-1 (1 μM). **H** Lipid ROS, (**I**) ferrous iron levels, (**J**). and GSH levels were measured in LM3 cells and HepG2 cells. The results shown are representative of 3 independent experiments. Data are represented as mean ± SD of biological triplicates *P-value < *0.05*; **P-value < *0.01*; ***P-value < *0.001* by unpaired, 2-tailed Student’s t-tests.

We further assessed the biological functions of USP8 in LM3 and HepG2 cells using two non-overlapping siRNAs separately. The proliferation rate of HCC cells treated with USP8 siRNAs was significantly suppressed compared with that in control cells (Fig. 5A). USP8 depletion significantly inhibited the colony formation ability of HCC cells (Fig. 5B). Consistently, USP8 depletion inhibited DNA synthesis as evaluated by Edu incorporation assay (Fig. 5C, D). Transwell assay demonstrated that knockdown of USP8 dramatically decreased the invasion capacity of LM3 and HepG2 cells (Fig. 5E). Previous studies reported that Wnt/β-catenin pathway is associated with the tumorigenicity of HCC cancer stem cells, we then examined the role of USP8 in HCC stemness characteristics. It was found that USP8 depletion significantly reduced the oncosphere formation of LM3 and HepG2 cells (Fig. 5F). Recent study reported that Wnt/beta-catenin signaling inhibited cellular lipid ROS production and subsequently enhanced ferroptosis resistance in gastric cancer [25]. We treated LM3 and HepG2 cells with different concentration of Erastin. The cell viability analysis demonstrated that USP8 depletion increased the resistance to erastin-induced ferroptosis of LM3 and HepG2 cells (Fig. 5G). We then assessed erastin-induced ferroptosis with or without ferrostatin-1 (Ferr-1), an inhibitor of ferroptosis. It is found that erastin-induced cell death could be rescued by ferr-1 in USP8 depleted-cells (Fig. 5H). We also found that depletion of USP8 increased lipid ROS levels and ferrous iron levels, while decreased the GSH levels in LM3 and HepG2 cells (Fig. 5I–K). We also observed that depletion of USP8 decreased the protein levels of GPX4, suggesting that USP8 may participate in ferroptosis through Wnt/beta-catenin/GPX4 axis (Fig. S1). To address the role of USP8 in HCC in vivo, we utilized a xenograft model to assess tumor formation in nude mice. Our results indicated that knockdown of USP8 or administration of erastin significantly inhibited tumor growth in vivo, erastin treatment and USP8 depletion in combination significantly inhibited tumor growth in vivo than administration of erastin or USP8 depletion alone (Fig. 5L). We also tested the effect of pharmacological USP8 inhibitor, DUB-IN-3 (0, 0.1, 0.5 μM), on the proliferation, invasion, stem-like properties and ferroptosis of HCC cells. As shown in Fig. 6, inhibition of USP8 by DUB-IN-3 caused a dose-dependent suppression on the proliferation, invasion, stem-like properties and promoted ferroptosis of HCC cells (Fig. 6A–J). As expected, DUB-IN-3 reduced β-catenin expression in a dose-dependent manner and decreased the half-life of β-catenin (Fig. 6K, L). Since USP8 deubiquitinates and stabilizes β-catenin in HCC cells, we then tested whether USP8 regulates these functions via modulating β-catenin. As shown in Fig. 7, the reconstitution of β-catenin efficiently rescued the effects induced by USP8 depletion. Our results suggested that USP8 promotes tumor progression by regulating β-catenin.

## Discussion

HCC is an aggressive and virulent solid tumor. The underlying molecule mechanisms involved in the initiation and progression of HCC remain largely unclear. It is an urgent issue to find out more novel candidate targets and improve the treatment decisions. Ubiquitination is one of the most important posttranslational modifications, it is essential for cellular homeostasis and plays a central role in the cellular protein-degradation machinery [26]. Ubiquitination involves the sequential transfer of an ubiquitin molecule mediated by three enzymes: ubiquitin-activating enzyme (E1), an ubiquitin-conjugating enzyme (E2) and an ubiquitin ligase (E3) [27]. The E3 ubiquitin ligases selectively mediate the ubiquitin conjugation of substrates, which could be reversed by deubiquitinating enzymes [28]. Dysregulation of E3 ligases or deubiquitinating enzymes is frequently observed in various human cancers. However, the potential roles of DUBs in HCC are not well established.

β-catenin activity plays an important role in regulating tumorigenesis. Accurate regulation of β-catenin is important for maintaining the functions of normal cells. Growing studies have reported that the protein stability of β-catenin can be regulated through the Ub-mediated proteasomal degradation. For instance, β-catenin can be phosphorylated and subsequently recognized by the E3 ubiquitin ligase β-TrCP, which promotes the formation of K48-linked polyubiquitin chains on β-catenin, resulting in the proteasomal degradation of β-catenin [29]. The linear ubiquitin ligase promotes linear ubiquitination of β-catenin in unstimulated cells, thus promoting its proteasome-dependent degradation [30]. DUBs also regulate the protein stability of β-catenin protein in human cancers cells. Upon DNA damage, the linear ubiquitination of β-catenin mediated by linear ubiquitin ligase could be reversed by OTULIN, which inhibits the proteasomal degradation β-catenin. OTULIN-mediated Wnt/β-catenin activation promotes metastasis and drug resistance of breast cancer cells [30]. The protein expression of β-catenin is positively associated with USP20 in patient samples and various cancer cell lines. In addition, USP20 depletion promotes the polyubiquitination of β-catenin, which facilitates β-catenin turnover and increases cell sensitivity to chemotherapy [31]. USP47 is identified as a DUB that prevents β-catenin ubiquitination. USP47 deubiquitinates and stabilizes β-catenin to regulate human cancer cell proliferation [32]. USP2a is reported as a DUB that can stabilize β-catenin through deubiquitination. USP2a promotes the nuclear accumulation of β-catenin and enhances its transcriptional activity, which facilitates the transcription of Wnt/β-catenin target genes [33].

To further systematically evaluate the impact of the individual members of the DUB family in β-catenin deubiquitination and tumor progression, we have screened a DUB siRNA library and conducted unbiased siRNA screening by monitoring the levels of β-catenin and identified several candidate DUBs. In addition to USP8, we also noticed a few other DUBs with potential effect on β-catenin signaling. However, siUSP47, siUSP2a, siUSP20, or siOTULIN imposed minimal effect on β-catenin in our screening systems. The seemingly contradicting results may arise from the variation of cellular context in different cell lines.

USP8 is a DUB that belongs to the ubiquitin-specific processing (USP) protease family. The expression of USP8 is frequently overexpressed in multiple cancer types, including lung cancer, breast cancer, cholangiocarcinoma, gastric cancer and melanoma [34–38]. USP8 is identified as a proliferation and metastasis enhancer in tumors [39]. Patients with high USP8 expression have shown worse prognosis [40]. A recent study reported that USP8 could reshape an inflamed tumor microenvironment (TME) to decrease the efficacy of anti-PD-L1/PD-1 immunotherapy. USP8 Inhibition enhances protein expression of PD-L1 by increasing the TRAF6-mediated Lys63-linked polyubiquitin chains of PD-L1 to antagonize Lys48-linked ubiquitination and degradation of PD-L1. Moreover, inhibition of USP8 activates the NF-κB signaling to triggers MHC-I expression and innate immune response. In several murine tumor models, USP8 inhibitor combination with anti-PD-L1/PD-1 immunotherapy effectively inhibits tumor growth and improves the survival benefit though increasing the infiltration of CD8 + T cells. [41]. In breast cancer, the type II TGF-β receptor TβRII is directly deubiquitinated and stabilized by USP8, which increases the expression of TβRII in the plasma membrane as well as tumor-derived extracellular vesicles (TEVs). USP8 also promotes epithelial-mesenchymal transition (EMT), invasion, and metastasis of tumor cells in response to TGF-β/SMAD signaling. High expression of USP8 enables TβRII + circulating extracellular vesicles to induce exhaustion of T cell and chemoimmunotherapy resistance [42]. The combination of USP8 inhibitor and PD-1/PD-L1 blockade may be potential therapeutic strategy for enhancing anti-tumor efficacy.

In the present study, we identified USP8 as a potent DUB which could deubiquitinate and stabilize β-catenin in HCC. First, USP8 and β-catenin interacted with each other. Co-IP analysis identified the interaction between USP8 and β-catenin. Second, USP8 decreased β-catenin polyubiquitination and promotes β-catenin protein stabilization in a manner that depending on its DUB activity. Deletion of USP8 markedly reduced the protein abundance of β-catenin, and the reduced β-catenin protein abundance could be restored by ectopic expression of USP8-WT, but not its catalytically inactive mutant USP8^C786A^. Under the treatment of the proteasome inhibitor MG132, USP8 deletion could not further affect β-catenin protein level. USP8 depletion shortened the half-life time of β-catenin protein. Ectopic expression of USP8-WT, but not USP8^C786A^, significantly decreased the ubiquitylation of β-catenin. In vivo deubiquitylation assays demonstrated that the ubiquitin chain on β-catenin could be directly removed by USP8 in a dose-dependent manner. To further find out which type of ubiquitin chain on β-catenin was removed by USP8, we performed ubiquitination assay using a series of ubiquitin mutants, including K6, K11, K27, K29, K33, K48, and K63. We observed that USP8 significantly decreased K48-linked polyubiquitination from β-catenin. As polyubiquitination through K48 of Ub generally results in proteasomal degradation [43, 44], USP8 may maintain the stability of β-catenin by removing the K48-linked ubiquitin chain from β-catenin protein. Finally, USP8 could promote tumor proliferation, invasion and stem-like properties of HCC through β-catenin. Knockdown of USP8 significantly inhibited tumor proliferation, invasion, and stem-like properties. In addition, the restoration of β-catenin expression abolished the effects induced by USP8 depletion. We further examined DUB-IN-3, a small molecular inhibitor of USP8, to verify our findings. As expected, DUB-IN-3 treatment suppressed HCC cell proliferation, invasion, and stem-like properties in a dose-dependent manner. Correspondingly, DUB-IN-3 treatment reduced the β-catenin protein abundance and decreased the polyubiquitination of β-catenin. CHX analysis indicated that the half-life of β-catenin was significantly shortened upon DUB-IN-3 treatment, suggesting that DUB-IN-3, like USP8 depletion, promotes β-catenin ubiquitination and degradation.

Ferroptosis is a newly defined form of programmed cell death, which is caused by the accumulation of reactive oxygen species (ROS), particularly lipid ROS [45–47]. ROS is physiologically produced by aerobic cells, an excess formation of ROS reacts with polyunsaturated fatty acids in the lipid membranes, inducing lipid peroxidation that leads to cell death. In order to prevent the irreversible damage, cells develop adaptive response to restore the redox homeostasis. GSH is an important scavenger of ROS. GSH level is increased in various types of tumors, elevated GSH level is essential for cell cycle progression and is associated with cellular proliferation and metastatic activity [48]. GSH reduces free radicals and decreases ferroptosis when it is reduced to GSSG by glutathione peroxidase-4 (GPX4) [49]. Depletion of GSH leads to overwhelming lipid peroxidation, ultimately the induction of ferroptosis. GPX4 is a kind of GSH-dependent reductase, which inhibits ferroptosis by decreasing the lipid peroxidization in cells [50]. GPX4 activity is considered as the key biomarker to directly monitor ferroptosis. Decreased intracellular GPX4 activity or direct degradation of GPX4 can lead to increased iron-dependent reactive oxygen species, then induce ferroptosis [51, 52]. Wnt/β-catenin signaling is a conserved signaling axis that participates in diverse physiological processes of tumor, it is one of the most attractive targets for cancer therapy. A recent study revealed that Wnt/beta-catenin signaling pathway attenuates the levels of cellular lipid peroxidation by upregulating the expression of GPX4, resulting in the inhibition of ferroptosis [25]. In the present study, we observed that inhibition of USP8 promoted ferroptosis of HCC cells. Further mechanism analysis indicated that USP8 stabilized β-catenin and depletion of USP8 decreased the protein levels of GPX4, suggesting that USP8 may participate in ferroptosis through Wnt/beta-catenin/GPX4 axis.

**Fig. 8:**
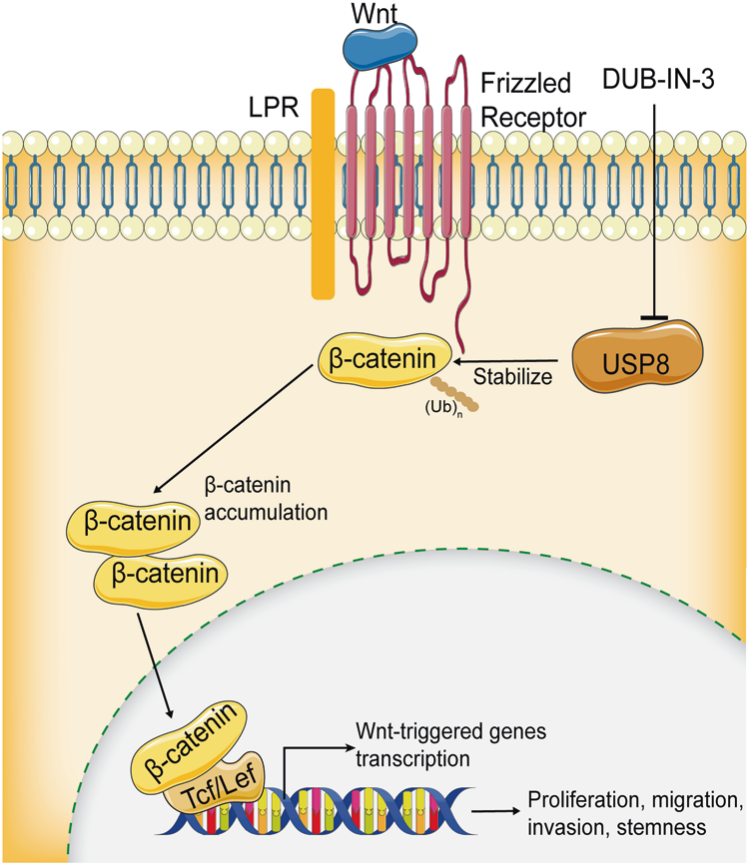
Mechanism diagram. USP8 is a β-catenin DUB that stabilizes β-catenin and promotes tumor growth, invasion, tumor stem-like properties, and ferroptosis resistance though its deubiquitylation activity.

In conclusion, we demonstrated that USP8 is a β-catenin DUB that stabilizes β-catenin and promotes tumor growth, invasion, tumor stem-like properties and ferroptosis resistance though its deubiquitylation activity (Fig. 8). Our findings provide new insights into the roles of USP8 in Wnt/β-catenin signaling pathway, and reveals a potential therapeutic strategy to utilize a USP8 inhibitor for HCC treatment.

## Materials and Methods

### Cell culture

The human HCC cell lines LM3, HepG2, and human embryonic kidney HEK293T cells were purchased from the Procell Life Science&Technology Co, Ltd (Wuhan, China). LM3, HepG2, and HEK293T cells were maintained in Dulbecco’s modified Eagle’s medium (DMEM, 41965, Life Technologies) supplemented with 10% fetal bovine serum (FBS, Gibco, Life Technologies, 10270). All cells were cultured at 37 °C, in 5% CO_2_ humid atmosphere.

### Plasmids and RNA inference

The Ubi and the mutant plasmids were described in our previous study [53]. Wild type (WT) and mutant USP8 or β-catenin plasmids were obtained from Hanbio Biotechnology Co., Ltd. (Shanghai, China). The Small interfering RNAs (siRNAs) targeting USP8 (siRNA-1: 5′- GGCAAGCCAUUUAAGAUUA-3′; 5′- CCACUAGCAUCCACAAGUA-3′) were obtained from Genepharma (Shanghai, China).

### RNA extraction and qRT-PCR analysis

Hipure total RNA mini kit (Magen, Guangzhou, China) was used to isolate the total RNA from the cancer cells. Reverse transcription was performed using the RevertAid First Strand cDNA Synthesis Kit (Thermo, Lithuania) according to the instructions. The SYBR green mix (Toyobo, Japan) and 7500 Fast Real-Time PCR System (Applied Biosystems, Singapore) were used for qRT-PCR analysis. Primers were listed as follows: GAPDH (forward: 5′-ACGGGAAGCTTGTCATCAAT-3′, reverse: 5′-TGGACTCCACGACGTACTCA-3′); β-catenin (forward: 5′- AAAGCGGCTGTTAGTCACTGG-3′; reverse: 5′-CGAGTCATTGCATACTGTCCAT-3′); CCND1 (forward: 5′-CCCTCGGTGTCCTACTTCAA-3′; reverse: 5′-GTGTTCAATGAAATCGTGCG-3′); c-MYC (forward: 5′-CCTTTGGGCGTTGGAAACC-3′; reverse: 5′- CGTCGCAGATGAAATAGGG-3′); LGR5 (forward: 5′-CAGCGTCTTCACCTCCTAC-3′; reverse: 5′-TTTCCCGCAAGACGTAACTC-3′).

### Luciferase assay

The TOP luciferase reporter plasmid and Renilla-TK-luciferase vector were obtained from Hanbio Biotechnology Co., Ltd. (Shanghai, China). Luciferase assay was performed by using Promega luciferase assay kit (E1910, WI, USA) according to the manufacturer’s instructions. HCC cells cultured in 24-well plates for 24 h were co-transfected with TOP luciferase reporter (0.2 µg), Renilla reporter (0.05 µg), and siControl or siUSP8 (50 nM). At 48 h post-transfection, luciferase activities were determined using a microplate reader (Varioskan LUX, Thermo Scientic). The ratio of reporter plasmids was determined, each normalized to the luciferase activities of the Renilla-TK-luciferase vector.

### Co-immunoprecipitation assay

Cells were washed with pre-chilled phosphate-buffered saline (PBS) and lysed with RIPA extraction reagent (Meilun, Dalian, China) supplemented with protease inhibitors (Meilun, Dalian, China). Cell lysates were pre-cleared and incubated with indicated antibody overnight at 4 °C, The antibody associated with the protein complex were then incubated with protein A/G PLUS-Agarose beads (beyotime, China) for additional 2 h. The beads were washed with PBS three times and boiled at 100 °C for 10 min to reverse crosslinking before SDS-PAGE immunoblotting analysis.

### Protein stability assay

To measure the half-life of β-catenin, cells were treated with 10 µg ml^−1^protein synthesis inhibitor cycloheximide (66-81-9, MCE) for indicated times. Western blot was performed to measure protein levels.

### In vivo deubiquitination assay

For in vivo deubiquitination assay, HA-Ub, Myc-β-catenin, Flag-USP8 or Flag-USP8 ^C786A^ plasmid were transfected into HEK293T cells for 48 h. Cells were then treated with 10 μM MG132 (133407-82-6, MCE) for 6 h. Then cells were washed with pre-chilled phosphate-buffered saline (PBS) and lysed with RIPA extraction reagent. HA-ubiquitinated β-catenin was isolated using an anti-Myc antibody. The ubiquitination level of β-catenin was detected by Western blotting with an anti-HA antibody. In HCC cells, HA-Ub plasmid and USP8 siRNAs were co-transfected into LM3 cells. HA-ubiquitinated β-catenin was isolated using an anti-β-catenin antibody. The ubiquitination level of β-catenin was detected by Western blotting with an anti-HA antibody.

### Western blot analysis

NP-40 lysis buffer supplemented with protease inhibitors (MB2678, Meilun, Dalian, China) was used to extract total protein. BCA Reagent (Thermo Scientific, Rockford, IL, USA) was used to measure the protein concentration. We used an SDS-polyacrylamide gel was to separate the total proteins and transferred proteins to 0.45 μm PVDF membrane (Millipore, USA). The primary antibodies used for western blot analysis are listed as follows: β-catenin (Proteintech, 17565-1-AP, Wuhan, China), USP8 (Proteintech, 67321-1-Ig, Wuhan, China), GAPDH (Proteintech, 60004-1-Ig, Wuhan, China), Myc (Proteintech, 60003-2-Ig, Wuhan, China), HA (Proteintech, 51064-2-AP, Wuhan, China), Flag (66008-4-Ig, Wuhan, China) antibodies. Signals were detected and visualized using ECL (MA0186, Meilun, Dalian, China) and ChemiDocMP imager (Bio-Rad).

### Cell proliferation analysis

For cell proliferation assay, LM3 and HepG2 cells were seeded in 96-well culture plates (2000 cells per well). The proliferation rate of HCC cells was detected using Cell Counting Kit-8 (CCK8, MA0218, Meilun, Dalian, China) assay at 0 h, 24 h, 48 h, 72 h, and 96 h according to the manufacturer’s instructions. The absorbance of each well was detected at a wavelength of 450 nm (SpectraMax M5, Molecular Devices, US). For clone formation assay, LM3 and HepG2 cells were seeded into 6-well plates (1000 cells per well) and incubated for 14 days, cells were washed with PBS and stained with 0.5% crystal violet. EdU (Ribobio, Guangzhou, China) incorporation assay was performed as we previously reported [54]. Briefly, cells were culture in 96-well plates at a density of 1000 cells per well. After 24 h, 50 μM EdU was added to each well and incubated for additional 2 h. The cells were fixed with 4% formaldehyde for 15 min and treated with 0.5% Triton X-100 for 20 min. After washing with PBS, 100 μl of 1 × Apollo reaction cocktail was added and incubated for 30 min. After staining with 100 μl of Hoechst 33342 for 30 min, the cells were visualized using EVOS cell image system.

### Cell invasion analysis

We performed transwell invasion assay using 8 μm pore polycarbonate membrane transwell plates (Corning, USA). Briefly, 5 × 10^5^ cells were suspended without serum and were cultured into the upper compartment of a transwell insert precoated with Matrigel.. The bottom chambers were filled with 600 μl complete medium. After 24 h, the invasion cells were fixed and visualized by crystal violet staining.

### Sphere formation assay

2 × 10^3^ single cells were seeded into 6-well ultra-low attachment culture plates (Corning, USA) in serum-free DMEM/F12 (Gibco, Life Technologies, 11320) supplemented with 20 ng/ml EGF (CM120, Tsingmu Biotechnology, Wuhan, China), B27 (1:50, 12587010, Gibco), and 20 ng/ml bFGF (CM091, Tsingmu Biotechnology, Wuhan, China). Two weeks later, the spheres were photographed and counted.

### In vivo tumorigenesis assay

Animal experiments were conducted according to the protocols approved by ethnic committee of Xiangya Hospital. 1 × 10^6^ LM3 cells were resuspended in 100 μl DMEM and injected subcutaneously into the flanks of BALB/c nude mice aged 6 weeks (*n* = 6). Mice were treated with vehicle or erastin (15 mg per kg intraperitoneal, three times a week) when the tumor volume reached 50 mm^3^. We used a vernier caliper to measure the tumor sizes and recorded every 5 days until the end of the experiment.

## Supplementary information

Reproducibility checklist

supplementary Figure
