# Supplementary figures and images for: USP8 positively regulates hepatocellular carcinoma tumorigenesis and confers ferroptosis resistance through β-catenin stabilization
Source: Cell Death Dis. 2023 Jun 13;14(6):360. doi: 10.1038/s41419-023-05747-7 (PMC10264414; doi:10.1038/s41419-023-05747-7)

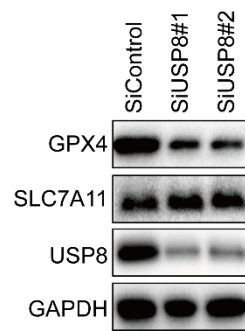

**Figure S1.** Depletion of USP8 decreased the protein levels of GPX4.

Supplement: Supplementary file 2 — supplementary Figure [file 41419_2023_5747_MOESM2_ESM.pdf]
